# Climate concerns for clinicians: evaluating harmful algal bloom knowledge and educational opportunities for health care provider students

**DOI:** 10.3389/fmed.2025.1597926

**Published:** 2025-11-04

**Authors:** Alexander Lund, Carrie McNair, Chelsea McGowen, Janel Lowman, Robert Sobol, Michael Parsons, Jennifer Pierce

**Affiliations:** ^1^College of Medicine, The University of South Alabama, Mobile, AL, United States; ^2^Mitchell Cancer Institute, University of South Alabama, Mobile, AL, United States; ^3^Brown University, Providence, RI, United States; ^4^The Water School, Florida Gulf Coast University, Fort Myers, FL, United States

**Keywords:** harmful algal blooms, ciguatera, ciguatera poisoning, health professional education, clinical training, medical students, PA students, climate change

## Abstract

With increasing incidence of harmful algal blooms (HABs) and their associated illnesses such as ciguatera poisoning (CP), there is need for educating current and future clinicians. This study sought to assess medical and physician assistant (PA) students' knowledge, attitudes, and beliefs toward HABs and their related illnesses. A survey of medical students and PA students at the University of South Alabama (USA) was conducted using an online questionnaire on climate change, HABs/associated illnesses, and CP. Response rate was calculated using fully executed questionnaires. Frequency data was utilized for demographics and knowledge-based questions; stratified analysis was used for associations. Three hundred three medical students received the questionnaire; 27% (*n* = 81) completed it. One hundred seventy-one PA students received the questionnaire; 19% (*n* = 33) completed the survey. These students were demographically representative of their student bodies. Out of 10 questions regarding knowledge of HABs, the percent correct was 30.7% for medical students and 20.3% for PA students. 34.6% of medical students and 47.1% of PA students had never heard of HABs. 90.1% of medical students and 84.8% of PA students believed climate change will impact human health in the future and more knowledge is needed about the relationship between health and climate change. Seventy six percentage of medical students and 51.6% of PA students expressed that HAB education should be part of health professional school curriculum. Future clinicians have little knowledge of HABs and their associated illnesses despite recognizing that climate change is a vital health issue. Medical and health professional schools should consider adding HAB education for future clinicians.

## 1 Introduction

The World Health Organization (WHO) has declared climate change as the single biggest threat to humanity (1). One effect of climate change has been warming ocean temperatures, leading to the increasing incidence of harmful algal blooms (HABs) with their attendant sequelae of aquatic and human poisoning incidents in the last century (2). Such sequelae include ciguatera poisoning (CP) which has a high incidence in areas surrounding the Caribbean and the Pacific (3). CP is caused by consuming tropical fish that fed on algae producing the neurotoxin ciguatera and is the most common seafood-borne illness worldwide, with around 15,000 cases per year in the United States (4). As the oceans warm, cases of CP are on the rise and growing more common outside of tropical locations in part due to climate change but also related to global food trade expansion. Currently, there is inefficient reporting for CP and the WHO predicts that climate change and international trade could increase the risk of CP (3). Given this, it is essential that future physicians and health professionals are trained to recognize common signs and symptoms in patients who have contracted CP. In 2000, family medicine physicians in Florida were tested with signs and symptoms of CP to elicit knowledge about diagnosis, management, and treatment (5). The study found that even in an endemic area, CP was underdiagnosed, inadequately treated, and under-reported, signifying that knowledge was lacking. A more recent survey of health care provider confidence in diagnosing HAB-related illnesses found that most reported little to no confidence in their ability, with most citing lack of knowledge as the reason (6).

With the increasing risk of CP and HAB-associated illnesses, there is an opportunity to ensure health professional students receive formal training on these conditions. Medical students are aware that climate change affects humans negatively and is a public health concern (7); however, there is a call to increase educational opportunities within medical school curricula (8). There have been increasing, though modest, efforts made by health professional schools to integrate educational modules regarding climate change into their curriculum (9). Surveys have been undertaken assessing students' knowledge and perceptions about climate change and global warming (10). Given the rise of HABs and CP without a concomitant increase in knowledge and education, there is a growing need to educate future and current clinicians. The purpose of this study was to assess the knowledge of and attitudes toward HABs and their related illnesses and ciguatera poisoning specifically of medical and physician assistant (PA) students attending a university in the Gulf of Mexico region of the United States.

## 2 Materials and methods

A cross-sectional survey was developed to assess medical and PA students' knowledge using an online questionnaire created in REDCap. Study data were collected and managed using REDCap electronic data capture tools hosted at the University of South Alabama (11, 12). REDCap (Research Electronic Data Capture) is a secure, web-based software platform designed to support data capture for research studies, providing (1) an intuitive interface for validated data capture; (2) audit trails for tracking data manipulation and export procedures; (3) automated export procedures for seamless data downloads to common statistical packages; and (4) procedures for data integration and interoperability with external sources. The questionnaire was divided into four sections: Part A assessed basic demographics and current stage of professional development; Parts B and C assessed knowledge about climate change, HABs, HAB-associated illnesses, and ciguatera poisoning; Part D assessed attitudes and beliefs regarding these topics plus education on them. Part B of the knowledge section included 10 multiple-choice questions, each with one correct answer. Of note, the possible answers for each knowledge question included “I do not know” as a response. In Part C of the knowledge section, participants were asked two questions about potential sources of information from which they might have heard of HABs and ciguatera and one question about which specific HABs they had heard about. These three questions had “check all that apply” lists of responses. The two questions about sources had “Other” as an option with a prompt to specify in short answer; the question about specific HABs included a response of “none of the above.” The final section, Part D, included 10 statements rated on a 5-point Likert scale from Strongly Agree to Strongly Disagree regarding beliefs and attitudes about HABs, climate change, and ciguatera poisoning.

This questionnaire was distributed to all medical students and PA students by mass email via the University of South Alabama (USA) College of Medicine's and College of Health Professions' registrar's offices respectively in July 2022. The survey remained open for 2 weeks, with a reminder email sent at 1 week. The response rate that was calculated used only participants who completed the survey in its entirety. Incomplete surveys were excluded from data analysis. Frequency data was used for analysis of demographics and knowledge-based questions. Stratified analysis was employed to look for associations and these were examined with logistic regression.

## 3 Results

Out of the 303 medical students who received the questionnaire, 29% (*n* = 88) opened it and 27% (*n* = 81) completed it. Out of the 171 PA students who received the questionnaire, 21% (*n* = 36) opened it and 19% (*n* = 33) completed the survey. The average age for both medical and PA students was 24.8 years. Half of the medical student respondents were in their second year with 3.4%, 28.4%, and 18.2% being first, third, and fourth-year students respectively. Of the PA students, 66.7% were in the didactic phase and 33.3% were in the clinical phase of training. Overall, more females completed the questionnaire at 62.5% for medical students and 94.4% for PA students. This was representative of the gender makeup of the medical and PA students' classes as reported by the registrar's offices. Most students who completed the questionnaire were white: 77.3% of medical students and 97.2% of PA students, also in line with reported class demographics. The rest of the medical student respondents were 9.1% Asian, 13.6% Black, 1.1% Native American, and 2.3% other. Other PA student respondents were 2.8% Black and 2.8% Native American. Ninety-two percentage of respondents' highest degree earned before medical school was a bachelor's degree and 88.9% of respondents' highest degree prior to PA school was a bachelor's degree; the remaining respondents had obtained a master's degree prior to enrollment.

**Figure 1:**
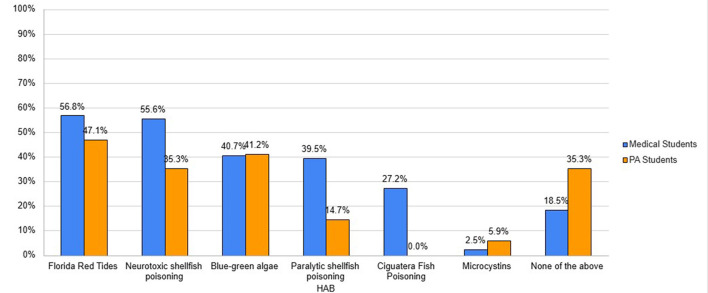
Student awareness of HAB and associated illnesses.

**Figure 2:**
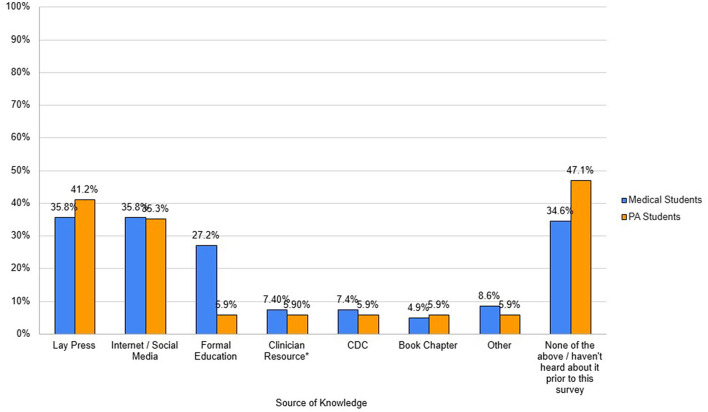
Source of knowledge about HABs.

Medical students had an average percent correct of 30.7% on the knowledge portion. Medical students answered 19.4% of the questions incorrectly and were unsure of 50% of the answers. PA students had an average percent correct of 20.3% on the knowledge section. They answered 22.1% of the answers incorrectly and were unsure of 57.6% of the answers. Regarding student awareness of HABs (Figure 1), 56.8% of medical students and 47.1% of PA students had heard about Florida Red Tides (the most common HAB in Florida and Alabama coastal waters) and 55.6% of medical students and 35.3% of PA students had heard about neurotoxic shellfish poisoning. An almost equal portion of medical students and PA students had heard of blue-green algae but there were stark differences in knowledge of paralytic shellfish poisoning (39.5% and 14.7% of medical and PA students), ciguatera poisoning (27.2% of medical students and 0.0% of PA students), and microcystins (2.5% and 5.9% of medical and PA students). Regarding the source of knowledge about HABs (Figure 2), out of the 65.4% of medical students that had heard about HABs, they had heard of them either from a combination of the lay press (35.8%), internet or social media (35.8%), or formal education (27.2%). About a third of medical students had not heard about HABs at all prior to the questionnaire (34.6%). About half (52.9%) of PA students who had heard of HABs had heard about them primarily from the lay press (41.2%) or the internet or social media (35.3%). About half of PA students reported they had not heard about HABs at all prior to the questionnaire (47.1%). Less than 10% of medical and PA students who had heard about HABs learned about them from a clinician resource: 7.4% and 5.9%, respectively from the CDC, 4.9% and 5.9% from a book chapter, and 8.6% and 5.9% from another clinical resource not listed in the questionnaire.

**Figure 3:**
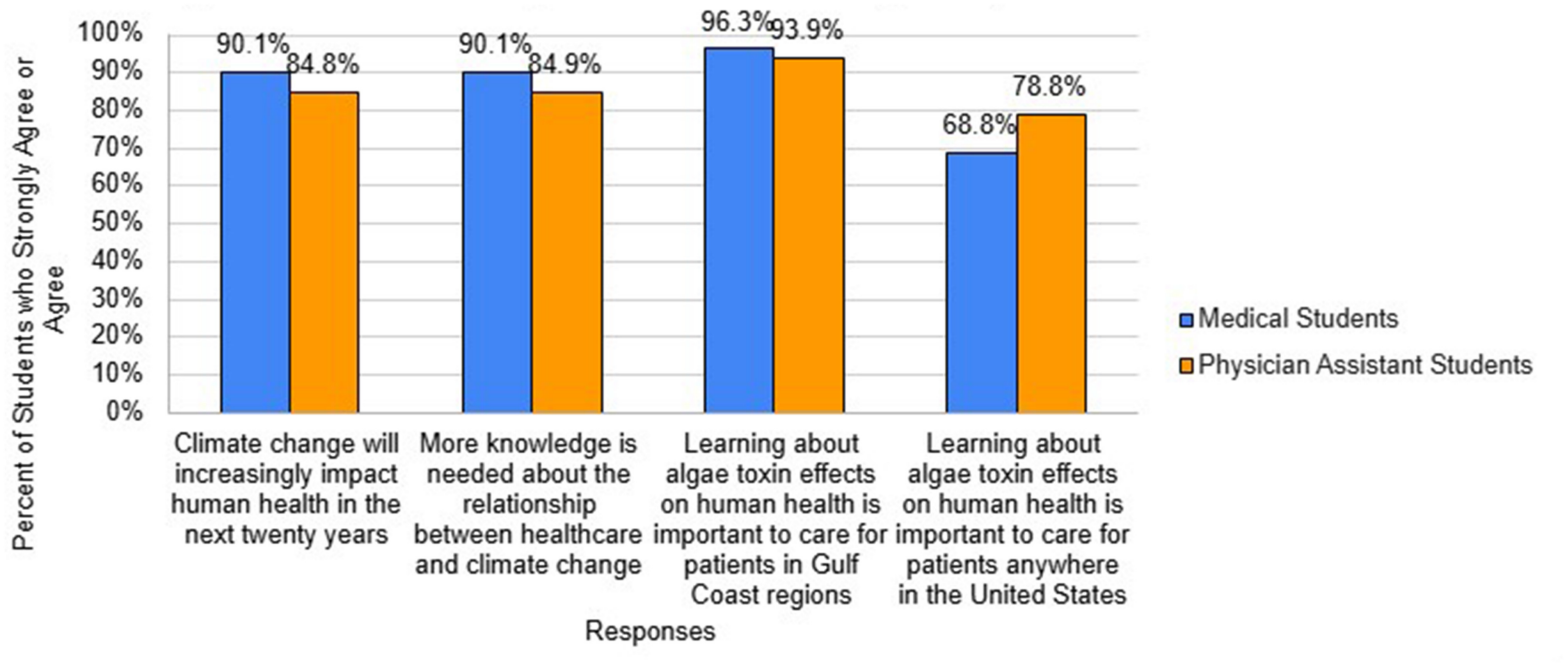
Student agree climate change impacts human health.

In assessing attitudes on climate change (Figure 3), 90.1% of medical students and 84.8% of PA students agreed that climate change will increasingly impact human health in the next 20 years. Similarly, 90.1% of medical students and 84.9% of PA students agreed that more knowledge is needed about the relationship between health care and climate change. The overwhelming majority of medical students (96.3%) and PA students (93.9%) agreed that learning about algal toxin effects on human health is important to care for patients in Gulf Coast regions and the majority (68.8% of medical students and 78.8% of PA students) also agreed it is important for care of patients anywhere in the United States.

**Figure 4:**
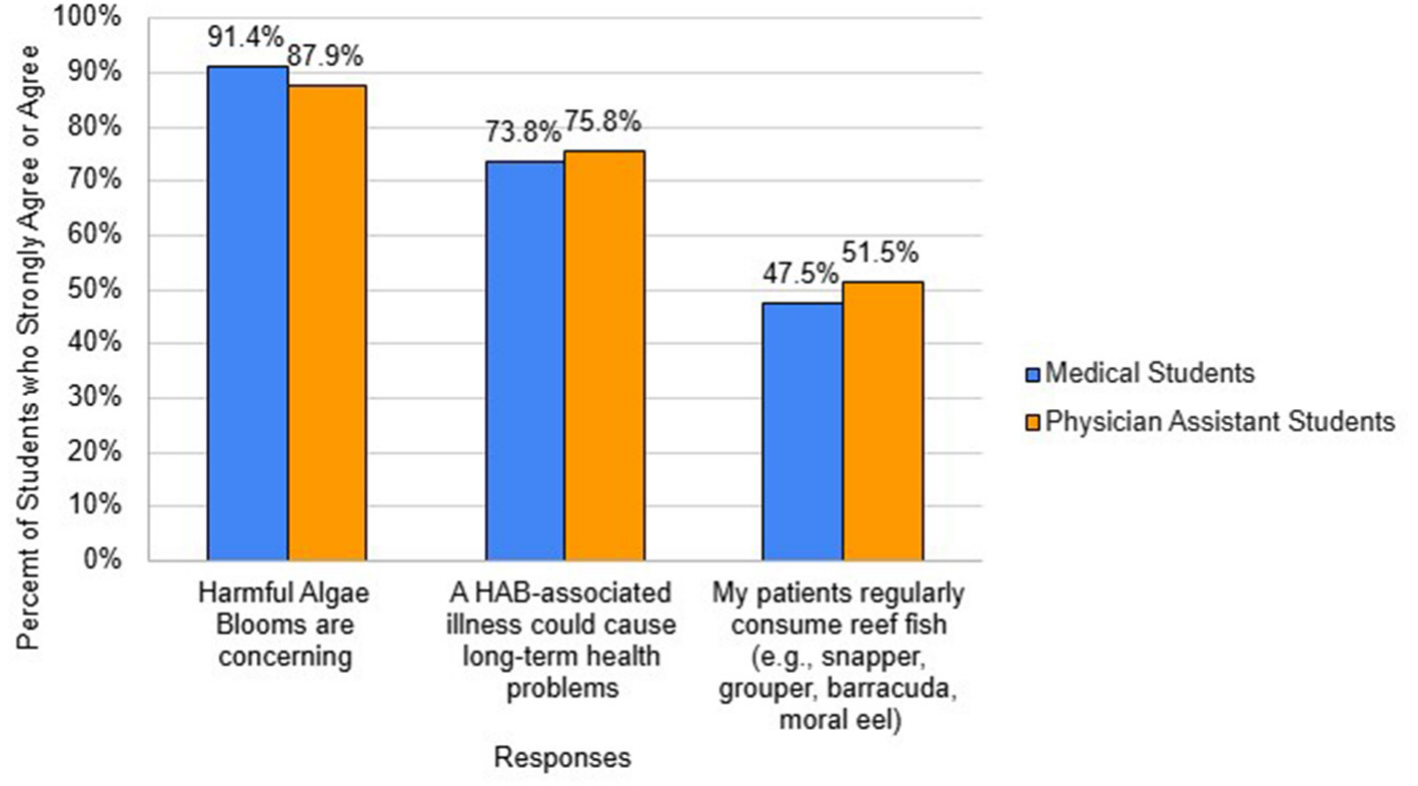
Beliefs to motivate action about HABs.

Regarding beliefs about the motivation to act on HABs (Figure 4), 91.4% of medical students and 87.9% PA students agree that HABs are concerning. Similarly, 73.8% of medical students and 75.8% of PA students agree that a HAB-associated illness could cause long term health problems. Approximately half of medical students and PA students agree that their patients regularly consume reef fish which are the types of fish most likely to be affected by ciguatoxins in endemic areas. In reference to beliefs concerning HAB education, 33.8% and 18.2% of medical students and PA students, respectively agree that learning about HABs will be time-consuming. 21.5% and 18.2% of medical students and PA students, respectively agree that learning about HABs would escalate their anxiety about them. However, 76.0% and 51.6% of medical students and PA students agree that HAB education should be a part of a health professional school's curriculum.

## 4 Discussion

In this survey health professional students demonstrated limited medical knowledge regarding HABs and ciguatera poisoning. Medical students on average answered only three out of 10 knowledge questions correctly with PA students answering just two out of 10 questions correctly. Students responding believed that climate change is an important issue that will impact human health and that more knowledge is needed. Notably, 96.3% and 93.9% of medical and PA students, respectively believe that learning about algal toxin effects is important for patient care in the north central Gulf of Mexico coast, the area of the United States in which the University of South Alabama is located.

This study utilized health behavior theories to inform questions and sought to understand students' knowledge, attitudes, and beliefs surrounding HABs. Whereas, health care providers have been surveyed on these topics, to our knowledge this is the first study to survey health professional students on climate change, HABs, and specifically ciguatera poisoning. This study attempts to quantify knowledge and perceptions of HABs and CP in a geographic area where they are on the rise. Further, this questionnaire includes ease of analysis with standardized answers being employed. Another strength was that this study was completed at a low cost and accessibility to the survey through email and REDCap made it easy to use and complete. A weakness of this study was the small sample size and low response rate, although the samples in both classes were demographically representative of the whole student body of each school, so no systemic under-sampling is suspected.

In recent history, harmful algal blooms and the impacts on human health have risen significantly (13) and continue to increase. However, this study suggests that health professional students are undereducated and inadequately prepared to manage patients with illnesses caused by HABs, like ciguatera poisoning, given the low percentage of respondents answering correctly in the knowledge section of the survey. Students' attitudes in this study indicate that they believe knowledge about HABs is important for patient care, especially in the Gulf Coast region and beyond, and that they are willing to take the time to include it in their formal education. Student respondents overwhelmingly agree that climate change impacts human health and that knowledge of the impact of climate change on human health will be important for their careers. This is consistent with a German study stating that their “final year” medical students recognized that global climate change affects the health of humanity (14). Additionally, they also concluded that there was a great opportunity for medical schools to introduce formal climate change education into the curriculum.

An opportunity is present for medical and health professional schools to begin incorporating not only climate change and its effect on human health into medical school curriculum, but also sequelae of climate change, namely harmful algal blooms and HAB-related illnesses. For example, ciguatera poisoning historically has been confined to isolated tropical islands with a heavy emphasis on fishing but due to the international seafood trade, travel, and changes in the aquatic environment, patients being sickened from ciguatera poisoning may be far away from where the fish was caught (15). In addition, ocean warming from climate change is projected to increase the growth and abundance of ciguatera-producing algae outside of the historically known areas which will change CP risks in the coming decades (16). The Greater Caribbean Center for Ciguatera Research (GCCCR) includes in its area of study the region in which University of South Alabama is located, in anticipation of climate change increasing the potential for ciguatera in the east and northern Gulf of Mexico (17).

Current research must aim to fill the knowledge gaps in climate change, HABs, and their effect on human health. Future research should consider focusing on best practices in medical school curriculums to educate health profession students regarding other diseases related to changing ocean environments due to climate change and their impact on human health. Health care professionals need to be armed with the knowledge to diagnose and care for these patients anywhere in the world. Though there are dedicated online sources of information on HABs and CP, such as the U.S. National Office for Harmful Algal Blooms and the CDC (18, 19), only about a third of students in this study said their HABs knowledge was from online sources. Dissemination of HABs and CP knowledge from a clinical standpoint should be part of formal training for health professionals while they are still students to give them working knowledge in their careers. The Association of State and Territorial Health Officials (ASTHO) has a clinical toolbox on cyanobacterial blooms and associated illnesses clinical toolbox for providers that could be used as continuing education but the opportunity is present to train students through their curriculum (20). Medical and PA students recognize a need and desire to learn more about HABs, climate change, and their effects on patients for whom they will be caring, and it is up to the medical education community to answer the call and provide this knowledge.

Despite recognizing that climate change is an important health issue, future health care providers have insufficient knowledge of harmful algal blooms and their associated illnesses. The ability to diagnose and treat ciguatera poisoning and other HAB-related illnesses is increasingly important, requiring formal education on these subjects. Adding HAB education to the curriculum of medical and health professional schools should be considered given the impact of climate change on human health.

## Data Availability

The raw data supporting the conclusions of this article will be made available by the authors, without undue reservation.
