# Supplementary material for: Climate concerns for clinicians: evaluating harmful algal bloom knowledge and educational opportunities for health care provider students
Source: Front Med (Lausanne). 2025 Nov 4;12:1597926. doi: 10.3389/fmed.2025.1597926 (PMC12623342; doi:10.3389/fmed.2025.1597926)
Supplement: Supplementary file 1 [file Supplementary_file_1.pdf]

Supplementary Material

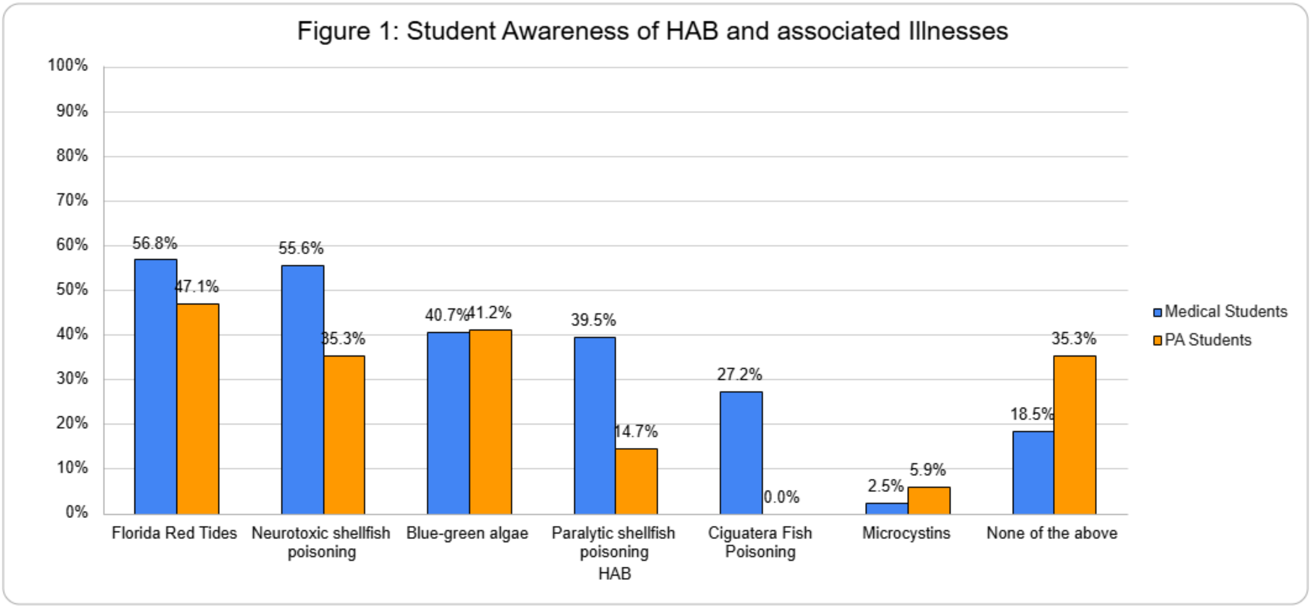

Figure 1. Percentage of survey respondents (n=81 medical students; n=33 PA students) answering that they had heard of these HABs and HAB-associated illnesses prior to taking the survey.

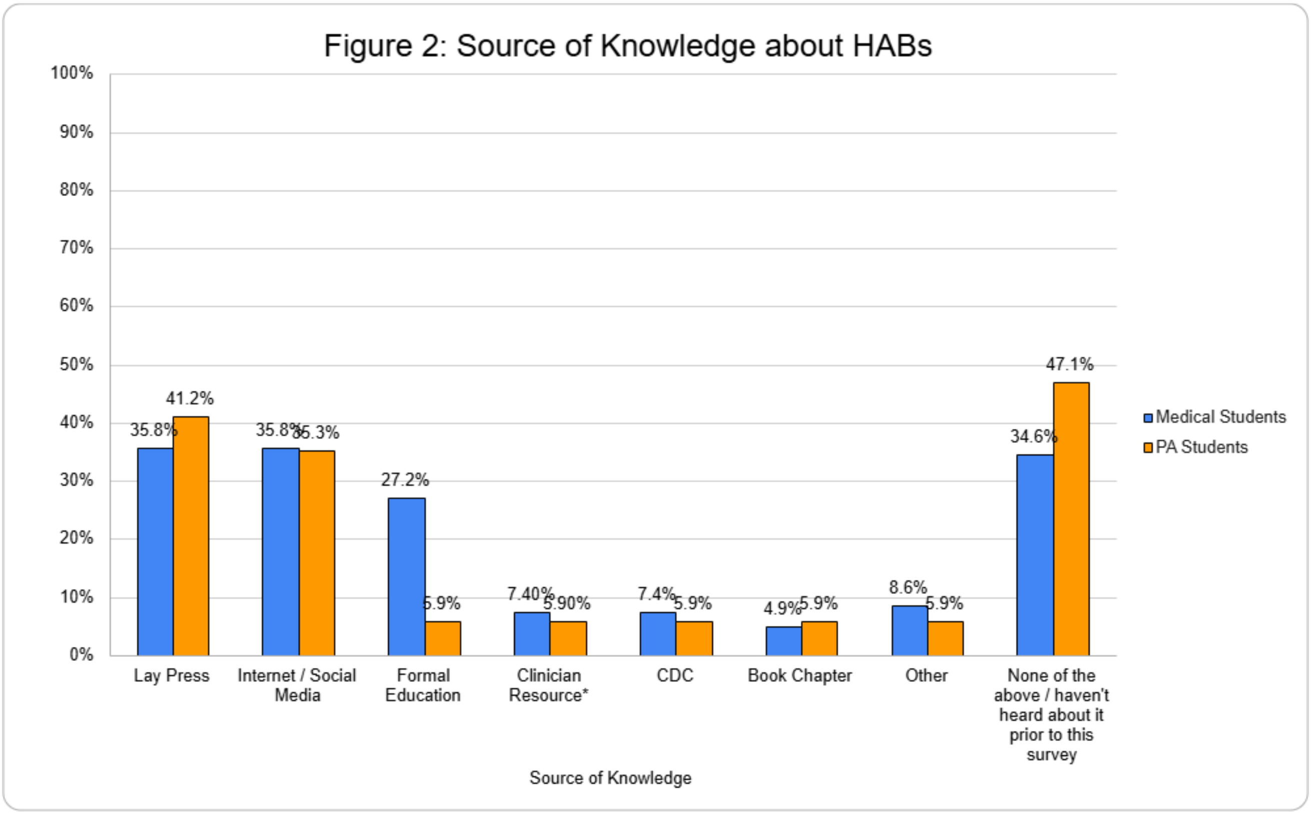

Figure 2: Percentage of survey respondents (n=81 medical students; n=33 PA students) indicating the sources of their knowledge of HABs and HAB-associated illnesses.

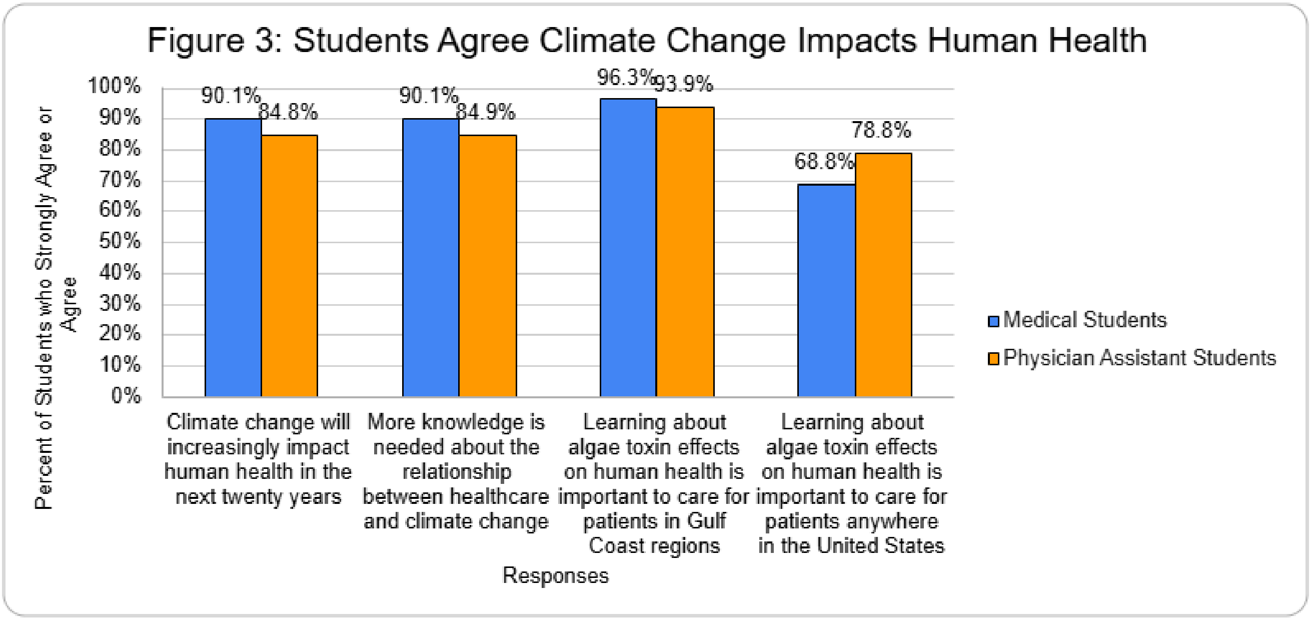

Figure 3: Percentage of survey respondents (n=81 medical students; n=33 PA students) who believe climate change impacts health and clinical knowledge of HAB-related illnesses is necessary.

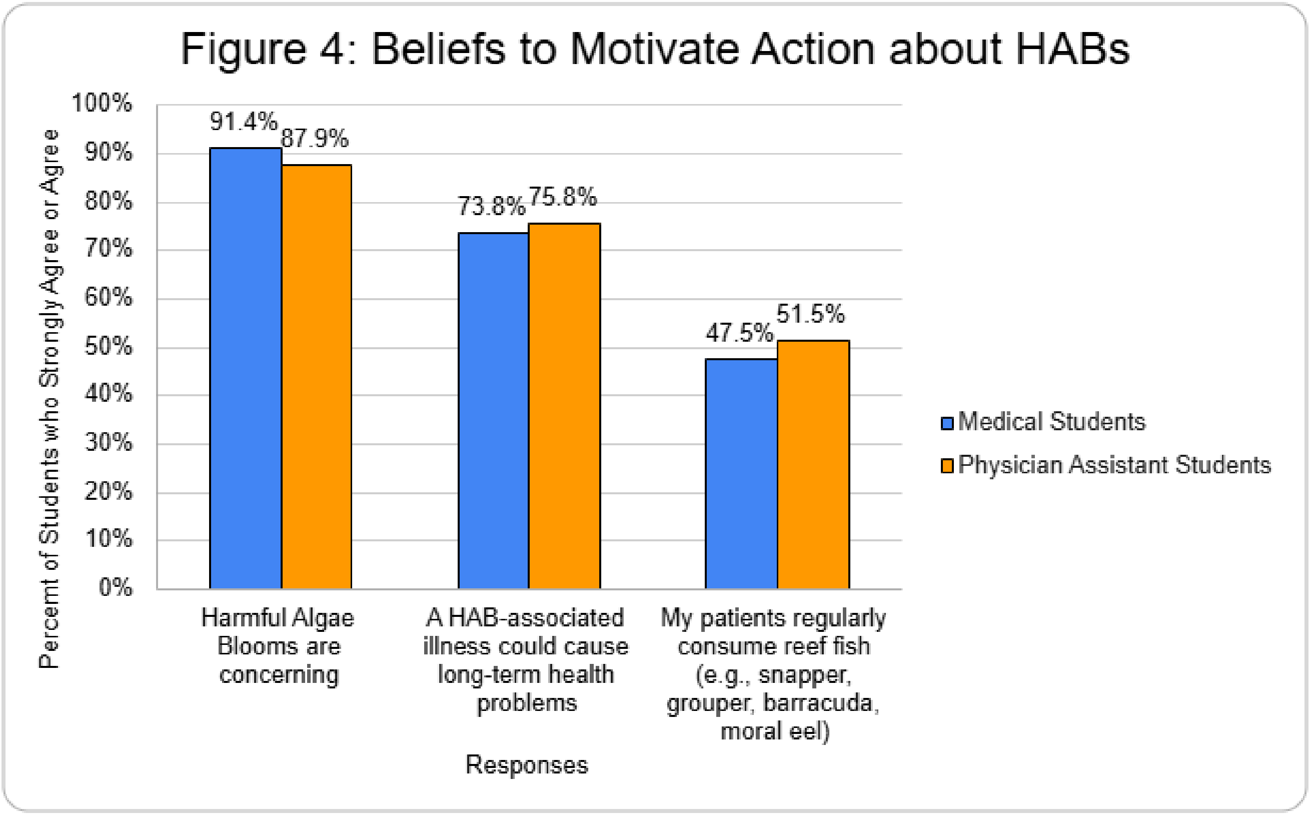

Figure 4: Percentage of survey respondents (n=81 medical students; n=33 PA students) who strongly agree or agree with concerns about HABs and risk of their patients.

Coastal Concerns for Clinicians: Evaluating harmful algae bloom knowledge and opportunities to increase provider education.

Principal Investigator: Alexander Lund

acl2122@jagmail.southalabama.edu

334-714-4491

You are invited to voluntarily participate in a research project regarding health professional students' and emergency medicine residents’ knowledge and attitudes toward harmful algae blooms and ciguatera fish poisoning.

The purpose of the study is to assess health professional students’ knowledge and emergency medicine residents’ harmful algae bloom knowledge using a questionnaire. This will take no longer than 10 minutes to complete. Participation will remain anonymous, and no identifying data will be collected.

Procedure: You will be asked to complete a questionnaire that first asks about your demographics. You will then be asked to answer knowledge questions that are in a multiple-choice format. Finally, you will be asked to select your level of agreement to statements that assess your attitude about harmful algae blooms. You have the right to refuse to answer any questions that you do not wish to complete and/or answer.

Benefits and Risks: Benefits include education and awareness of a topic with a growing interest in the scientific and medical community. Benefits could also be potentially advantageous in future society because of information gathered in the study. There are no risks to participating in this study.

Incentives: You will be included in a drawing of 100 dollars by Tango gift card for the completion of the harmful algae bloom and ciguatera questionnaire. The likelihood of being chosen is dependent on the number of participants and it is expected that 700 will be completed. The drawing will be conducted at Mitchell Cancer Institute in the presence of Dr. Jennifer Young Pierce on August 1st, 2022, at 2 P.M. You will be contacted by/through email if you have been selected.

Confidentiality: The data collected from your answers will be stored in REDCap and kept confidential. Only the research team will have access to the data. The only identifying information we will collect will be email addresses for the gift card drawing which will not be required to participate (you can submit the questionnaire without entering their email address into the drawing). Email addresses will be separated from survey responses and used for incentive drawing purposes only.

Voluntary Participation: Your participation in this research study is completely voluntary. You do not have to participate. You may quit at any time without any penalties.

You can withdraw at any time without consequence. Please contact me at 334-714-4491 or the Institutional Review Board at the University of South Alabama at (251) 460-6308 if you have questions about your rights as a research subject.

Part A: Demographics

What is your current age?

____

What is your gender?

- ◯ Male
- ◯ Female
- ◯ Transgender female
- ◯ Transgender male
- ◯ Other
- ◯ Prefer not to answer

If other, please specify:

____

What is your ethnicity?

- ◯ Hispanic or Latino
- ◯ Not Hispanic or Latino

What is your race? (check ALL that apply)

- ☐ Asian
- ☐ Black
- ☐ Native American (including Alaskan Native)
- ☐ Native Hawaiian or Pacific Islander
- ☐ White
- ☐ Other

If other, please specify:

____

Please select your role

- ◯ Medical Student
- ◯ Intern / Resident
- ◯ Physician Assistant student
- ◯ Nurse Practitioner student

What year in medical school are you?

- ◯ First year
- ◯ Second year
- ◯ Third year
- ◯ Fourth year
- ◯ Year out
- ◯ Not graduated / not actively enrolled
- ◯ Ph.D. part of MD-Ph.D.
- ◯ Other

If other, please specify:

____

What year in residency are you?

- ◯ PGY-1
- ◯ PGY-2
- ◯ PGY-3
- ◯ PGY-4
- ◯ PGY-5
- ◯ Fellow

What phase of PA training are you in?

- ◯ Didactic (pre-clinical)
- ◯ Clinical

What NP degree are you currently pursuing?

- ◯ MSN
- ◯ DNP
- ◯ Other

If other, please specify: __________________________________

What is the highest degree you obtained before health professional school?

- ◯ Bachelor's
- ◯ Master's
- ◯ PhD
- ◯ Other

Please specify which Bachelor's degree you obtained: __________________________________

Please specify which Master's degree you obtained: __________________________________

Please specify what your PhD degree is in: __________________________________

If other, please specify: __________________________________

Part B: Knowledge of harmful algae blooms and ciguatera poisoning

For each question, select the single best answer. If you do not know the answer, select "I do not know" rather than guessing an answer choice.

There are ten questions. After answering one, you will not be able to go back and change your answers.

1. Harmful algae blooms are characterized by:
   - ◯ Rapid growth of toxin-producing algae that harm other algae only
   - ◯ Rapid growth of toxin-producing algae that harm marine life only
   - ◯ Rapid growth of toxin-producing algae that can result in human, marine, or animal harm
   - ◯ Color changes in the water with no detectable harm
   - ◯ I do not know

2. In what way does a harmful algae bloom cause human illness?
   - ◯ Inhalation of algae bloom aerosols
   - ◯ Eating toxin-contaminated fish and shellfish
   - ◯ Skin contact with toxins produced from algae blooms
   - ◯ All of the above
   - ◯ None of the above, it is not harmful to humans
   - ◯ I do not know

3. Symptoms that are NOT typically characteristic of people affected by harmful algae blooms include:
   - ◯ Gastrointestinal symptoms (e.g., nausea, vomiting, diarrhea)
   - ◯ Neurologic symptoms (e.g., paresthesias, confusion, headache)
   - ◯ Dermatologic symptoms (e.g., skin rash, itchiness, irritation)
   - ◯ Urinary Symptoms (e.g., blood in urine, painful urination)
   - ◯ I do not know

4. Which one of the following is associated with harmful algae blooms?
   - ◯ Vibrio (one of the flesh-eating bacteria)
   - ◯ Poisonous seafood
   - ◯ Legionella
   - ◯ Brain-eating amoebas
   - ◯ I do not know

5. How could you protect yourself from eating a toxin caused by a harmful algae bloom?
   - ◯ Training chefs to identify toxic seafood
   - ◯ Cooking / steaming the seafood
   - ◯ Freezing fresh seafood prior to eating
   - ◯ By smelling specific odors in toxin-contaminated fish
   - ◯ Seeing if flies land on a toxic fish
   - ◯ None of the above
   - ◯ I do not know

6. Ciguatoxin is a type of toxin produced by harmful algae blooms. The main risk factor for acquiring ciguatoxin is:
   - ◯ Eating reef fish (e.g., snapper, grouper, barracuda, moray eel)
   - ◯ Eating shellfish (e.g., shrimp, crab, lobster, mussels, oysters)
   - ◯ Swimming in toxin-contaminated waters
   - ◯ Inhalation of small droplets of water in the air
   - ◯ I do not know

7. The main symptoms of ciguatera related illness are all EXCEPT:
   - ◯ Itchiness
   - ◯ Nausea, vomiting, diarrhea
   - ◯ Temperature hot-cold reversal (i.e., cold things feel hot)
   - ◯ Tingling/numbness
   - ◯ Paralysis
   - ◯ Hypotension
   - ◯ I do not know

8. Which is true regarding the diagnostics of ciguatera poisoning?
   - ◯ Symptoms mimicking an allergic reaction (e.g., skin flushing, warmth, rash) provide the diagnosis
   - ◯ History of ingestion of a large reef fish is essential for a definitive diagnosis
   - ◯ Nerve conduction studies are essential for a definitive diagnosis
   - ◯ I do not know

9. Which of the following is true regarding the treatment of ciguatera poisoning?
   - ◯ Stomach emptying with charcoal or syrup of ipecac is not necessary
   - ◯ Nothing can be done for the patient, as this is a fatal illness with no treatment options
   - ◯ Epinephrine needs to be given for the allergic reaction that has occurred
   - ◯ I do not know

10. The best supportive treatment for patients with neurologic symptoms in the absence of shock induced by ciguatera poisoning is thought to be:
    - ◯ Mannitol
    - ◯ Gabapentin
    - ◯ Lorazepam
    - ◯ I do not know

Part C: General Questions

In which of the following ways have you heard or read about harmful algae blooms (check ALL that apply)

- ☐ Lay press
- ☐ Medical news / medical journal
- ☐ UpToDate / Clinical Key
- ☐ Centers for Disease Control and Prevention (CDC)
- ☐ Book chapter
- ☐ Travel brochure/alert
- ☐ Internet / social media
- ☐ Formal education
- ☐ Other
- ☐ None of the above / haven't heard about it prior to this survey

Where did you learn about harmful algae blooms?

- ☐ As part of medical education
- ☐ Learned as a part of a nonmedical degree program (e.g., BS, MS, MPH, PhD, etc.)
- ☐ Conference / webinar
- ☐ On my own (e.g., self-study, life experiences, travel, etc.)
- ☐ Other

If other, please specify where you heard about them: ______________________

If other, please specify where you learned about them: ______________________

Which harmful algae blooms have you heard about prior to this survey? Check ALL that apply.

- ☐ Florida Red Tides
- ☐ Ciguatera Fish Poisoning
- ☐ Blue-green algae/cyanobacteria
- ☐ Microcystins
- ☐ Paralytic shellfish poisoning
- ☐ Neurotoxic shellfish poisoning
- ☐ None of the above

Part D: Attitude toward harmful algae blooms (HABs)

Please select your level of agreement with each statement

	Strongly Agree	Agree	Neutral	Disagree	Strongly Disagree
Climate change will increasingly impact human health in the next twenty years	○	○	○	○	○
More knowledge is needed about the relationship between healthcare and climate change	○	○	○	○	○
Harmful algae blooms (HABs) are concerning	○	○	○	○	○
Learning about algae toxin effects on human health is important to care for patients on the Gulf	○	○	○	○	○
Learning about algae toxin effects on human health is important to care for patients anywhere in the United States	○	○	○	○	○
My patients regularly consume reef fish (e.g., snapper, grouper, barracuda, moray eel)	○	○	○	○	○
A HAB-associated illness could cause long term health problems	○	○	○	○	○
Learning about HABs will be time-consuming	○	○	○	○	○
Learning about HABs will increase my anxiety about them	○	○	○	○	○
HAB education should be a part of a health professional school's curriculum	○	○	○	○	○
